# Gene- and Gender-Related Decrease in Serum BDNF Levels in Alzheimer’s Disease

**DOI:** 10.3390/ijms232314599

**Published:** 2022-11-23

**Authors:** Daniela Piancatelli, Anna Aureli, Pierluigi Sebastiani, Alessia Colanardi, Tiziana Del Beato, Lorenza Del Cane, Patrizia Sucapane, Carmine Marini, Silvia Di Loreto

**Affiliations:** 1 National Research Council (CNR)—Institute of Translational Pharmacology (IFT), 67100 L’Aquila, Italy; 2 Department of Life, Health and Environmental Sciences (MeSVA), University of L’Aquila, 67100 L’Aquila, Italy; 3 Neurology Unit, ASL1, San Salvatore Hospital, 67100 L’Aquila, Italy

**Keywords:** brain-derived neurotrophic factor (BDNF), Alzheimer’s disease, gender, oxidative stress, cytokines

## Abstract

Brain-derived neurotrophic factor (BDNF) has a protective role in Alzheimer’s disease (AD). Oxidative stress and inflammatory cytokines are potentially implicated in AD risk. In this study, BDNF was detected in serum of AD and mild cognitive impairment (MCI) patients and investigated in association with gene polymorphisms of BDNF (Val66Met and C270T), of some oxidative stress-related genes (FOXO3A, SIRT3, GLO1, and SOD2), and of interleukin-1 family genes (IL-1α, IL-1β, and IL-38). The APOE status and mini-mental state examination (MMSE) score were also evaluated. Serum BDNF was significantly lower in AD (*p* = 0.029), especially when comparing the female subsets (*p* = 0.005). Patients with BDNFVal/Val homozygous also had significantly lower circulating BDNF compared with controls (*p* = 0.010). Moreover, lower BDNF was associated with the presence of the T mutant allele of IL-1α(rs1800587) in AD (*p* = 0.040). These results were even more significant in the female subsets (BDNFVal/Val, *p* = 0.001; IL-1α, *p* = 0.013; males: ns). In conclusion, reduced serum levels of BDNF were found in AD; polymorphisms of the IL-1α and BDNF genes appear to be involved in changes in serum BDNF, particularly in female patients, while no effects of other gene variants affecting oxidative stress have been found. These findings add another step in identifying gender-related susceptibility to AD.

## 1. Introduction

Prevention and treatment of Alzheimer’s disease (AD) could benefit from the identification of specific biomarkers of disease risk, although these are still unknown. Neurotrophins, including brain-derived neurotrophic factor (BDNF), are a family of proteins that regulate memory and cognitive processes, synaptic plasticity, neuronal morphology, function, and survival [1]. Neurotrophins could be involved in the pathogenesis and progression of AD, as they have protective functions against neurodegenerative disorders and are found to be reduced in late stages and in more severe diseases [2].

It is known that BDNF levels are affected by lifestyle factors (diet, physical activity, etc.), medications, and gender-specific factors, such as hormones [3]. Lower levels of serum BDNF were previously reported in AD, in particular in severe cases, and described in meta-analyses [4,5]; at the same time, other studies reported higher or no changes in BDNF levels in AD [6,7].

Results of different studies about the implication of genetic polymorphisms of BDNF in AD and in other age-related diseases are still under investigation. The BDNF gene is located on chromosome 11p14.1; the most studied BDNF functional polymorphism consists of a nucleotide variation at position 196 in exon 5, which leads to a Val66Met substitution (rs6265). This mutation can cause a misfolding of the protein, with an impact on binding to TrkB receptor and on BDNF functions, mainly in the hippocampus [2] and on cognitive impairment in older subjects [8]. Significant associations between AD and BDNF polymorphisms, in particular Val66Met and C270T, have been reported, often with contradictory findings probably resulting from interactions with other factors (mainly gender, age, and ethnicity) that influence disease risk and progression [2,9].

Furthermore, polymorphisms of genes encoding for molecules involved in inflammation could affect disease onset and evolution; interleukin-1 (IL-1) genetic variations and haplotypes lead to different individual inflammatory responses and expression of inflammatory mediators. Increased levels of circulating molecules of the IL-1 family were found in AD [10,11] and IL-1 polymorphisms were associated with AD onset and pathogenesis [12]. However, it has been hypothesized that these gene variants should be considered not so much as causative factors, but rather as disease modifiers for AD; consequently, the possibility of modulating their effects opens up promising prospects [13].

Oxidative stress plays an important role in AD and mild cognitive impairment (MCI), and oxidative damage mainly affects the mitochondrial DNA [14,15]. Sirtuins (silent information regulator type, SIRT) are mitochondrial NAD ^+^ -dependent deacetylases implicated in the modulation of reactive oxygen species (ROS), in mitochondrial function and in aging; their polymorphisms were associated with neurodegeneration and AD. Superoxide dismutase 2 (SOD2) is an antioxidant enzyme that maintains the physiological control of the reactive oxygen species (ROS); its activity is enhanced by sirtuins. Glyoxalase-1 (GLO1), a detoxifying enzyme, is a part of the enzymatic defense system against methylglyoxal(MG)-induced glycation and is involved in protection from age-related diseases [16]. Recently, lower GLO1 levels were observed in AD and it was demonstrated that GLO1 is released from neuronal cells through extracellular vesicles (EV) [17], which are known to be involved in neurodegenerative diseases [18]. It has been hypothesized that gene variants of SIRT3 and Forkhead box O-3 (FOXO3A), a transcription factor highly expressed in the brain and involved in neuronal protection from oxidative stress [19], are related to longevity [20,21,22,23]. Both GLO1 and SOD2 polymorphisms have been associated with modifications of their enzymatic activity and increased susceptibility to oxidative stress [24]. Associations of the apolipoprotein (APO)E4 allele, a well-known risk factor for sporadic AD, and oxidative stress-related gene polymorphisms, in particular SOD2, may provide additional risk in AD and MCI [14,25].

In this study, relationships between serum levels of BDNF and some main gene polymorphisms of both BDNF (Val66Met, rs6265; C270T, rs56164415) and molecules potentially involved in inflammation (IL-1 family, including IL-1α rs1800587; IL-1β rs1143627; IL-38 rs6743376), in oxidative stress and mitochondrial damage or protection (APOE rs7412 and rs429358, FOXO3A rs2802292, SIRT3 rs11555236, GLO1 rs1049346 and SOD2 rs4880) were investigated in patients affected by AD or MCI, to determine potential predictive risk associations.

## 2. Results

### 2.1. Patients

As shown in Table 1, which describes the characteristics of patients included in the study, AD and controls did not differ significantly in age and gender composition, while the MCI group, as expected, consisted of significantly younger subjects (*p* = 0.002), with disease occurring more frequently before the age of 65 (*p* = 0.004).

Risk factors (head trauma, diabetes, smoking habits, early menopause) were observed in 24% of AD and 48% of MCI patients. The most common associated diseases were hypertension (AD 44%, MCI 58%), dysthyroidism (AD 11%, MCI 26%), and cardiovascular diseases (AD 16%, MCI 13%). The APOE polymorphisms (APOE4) were confirmed to be significantly associated with AD (*p* < 0.001) (Table 1, Table S1), although no significant associations were found with BDNF levels or with clinical and anamnestic characteristics.

### 2.2. Serum BDNF

**Figure 1:**
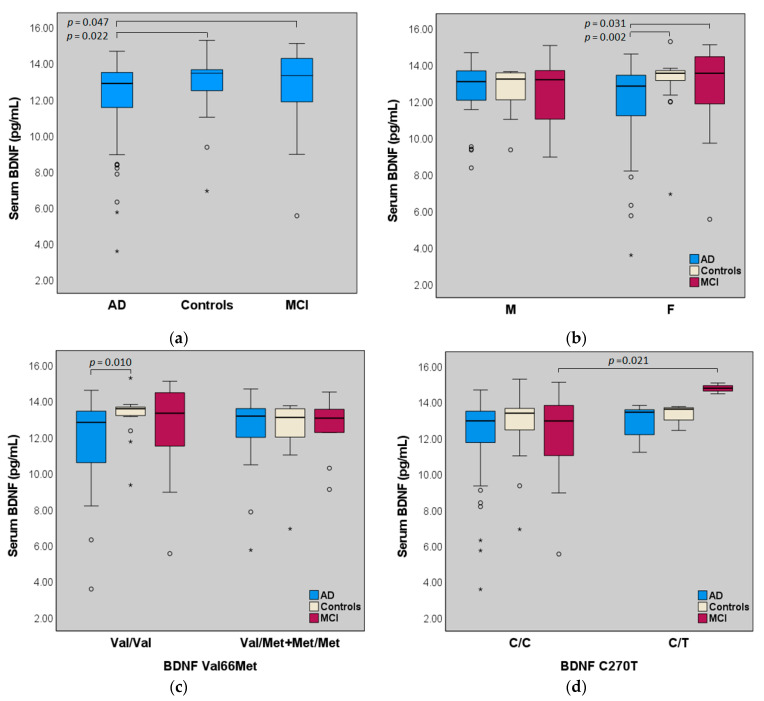
Box plot of serum BDNF (pg/mL) in AD (n  =  71), MCI (n = 31) and controls (n = 32). The upper line of the box marks the 75th percentile, the middle line is the median value, and the lower line represents the 25th percentile; whiskers above and below the box indicate the 90th and 10th percentiles, respectively; dots/stars indicate the outlier values within each group. (**a**) Serum BDNF in AD, MCI, and controls, *p* = 0.029 (Kruskal–Wallis test); (**b**) M/F clusters. In F, *p* = 0.005 between groups (Kruskal–Wallis test); (**c**) BDNF Val66Met genotype clusters. In Val/Val homozygous, *p* = 0.026 (Kruskal–Wallis test); (**d**) BDNF C270T genotype clusters. In C/T (n = 12), *p* = 0.035 (Kruskal–Wallis test); BDNF 270T/T homozygous were absent in all groups.

Serum BDNF was significantly different between groups (AD, MCI, and controls) (*p* = 0.029). It was reduced in AD, as compared with both MCI (*p* = 0.047) and controls (*p* = 0.022). (Figure 1a, Table 2). The presence of lower levels of BDNF in serum was more evident in females, while, in males, serum levels of BDNF were similar to that of the control group (Figure 1b) (females—AD vs. controls, *p* = 0.002; AD vs. MCI, *p* = 0.031; males—ns) (Table 2). Serum BDNF was also significantly reduced in females compared with males in severe AD (mini-mental state examination, MMSE, score under 20—females, n = 31, 11.25 ± 2.76 pg/mL; males, n = 13, 12.60 ± 1.65 pg/mL *p* = 0.049).

**Figure 2:**
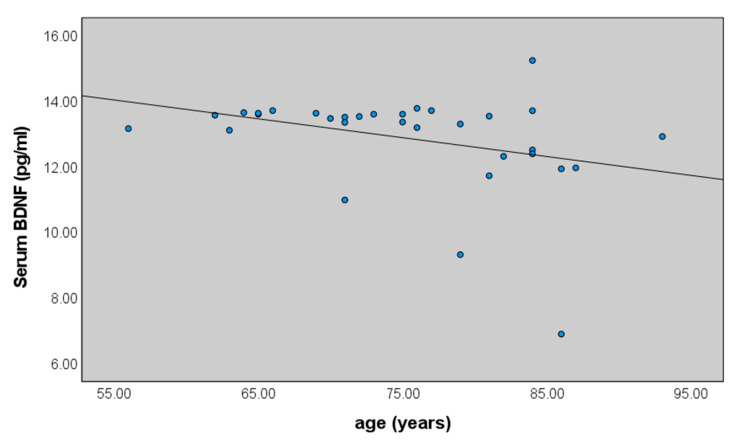
Correlation between serum levels of BDNF and age in controls (r = −0.377, *p* = 0.033).

No significant differences were detected between positive and negative family history and early vs. late onset diseases (AD or MCI). A weak correlation between BDNF and age was found only in controls (r = −0.377, *p* = 0.033). (Figure 2).

### 2.3. Alleles, Genotypes and Haplotypes

The Hardy–Weinberg equilibrium (HWE) of alleles was evaluated to test the hypothesis of a random union of gametes. All polymorphisms satisfied the HWE, as APOE rs7412 *p* = 0.345; APOE rs429358 *p* = 1.000; BDNF Val66Met *p* = 0.71198; BDNF C270T *p* = 1.000; SIRT3 *p* = 0.71156; SOD2 *p* = 0.79643; GLO1 *p* = 0.19621; FOXO3A *p* = 0.29273; IL-1α *p* = 1.00; IL-1β *p* = 0.594; IL-38 *p* = 1.00.

The association of APOE gene polymorphisms with AD was confirmed in the present study, as follows: a significantly different distribution of APOE genotypes and the ε4 allele between AD, MCI and controls was found (*p* = 0.001) (Table S1). The ε4 allele was more frequent in the AD cohort compared with controls (*p* < 0.0001, O.R. = 6.21, 95% C.I. 2.54–15.23).

Allele, genotype, and haplotype frequencies of the analyzed polymorphisms are shown in Figure S1 and Table S2; no significant differences were observed in allele, genotype, and haplotype frequencies between groups.

### 2.4. BDNF (Val66Met, C270T), IL-1α Gene Polymorphisms and Serum Levels of BDNF

No significant associations were found between serum levels of BDNF and gene polymorphisms involved in oxidative stress and mitochondrial damage or protection (APOE, FOXO3A, SIRT3, GLO1, and SOD2).

Due to the low frequency of the BDNF Met/Met homozygous, Met/Met plus Val/Met genotypes were analyzed together and compared with Val/Val carriers. No significant differences in serum levels of BDNF were detected between Val/Val and Met/Met plus Val/Met carriers in each group (AD, MCI, and controls, Figure 1, Table 2).

The significant difference of serum BDNF among groups (AD, MCI, and controls) was confirmed in carriers of BDNF Val/Val genotype (*p* = 0.026); patients with BDNF Val/Val homozygous had significantly lower serum BDNF levels (11.78 ± 2.40 pg/mL compared with Val/Val homozygous controls (13.21 ± 1.19 pg/mL *p* = 0.01), in particular when considering the female subsets (11.63 ± 2.66 pg/mL *p* = 0.001). In BDNF Met/Met + Val/Met carriers, no significant differences were detected between AD and controls (Figure 1c, Table 2) and according to gender (AD in females, n = 19, 12.11 ± 2.12 pg/ mL vs. males, n = 9, 13.17 ± 0.99 pg/mL *p* = ns).

The T/T homozygous of the BDNF C270T polymorphism were absent in all groups; the T/C carriers showed increased circulating BDNF, particularly in the MCI group (*p* = 0.021 between C/C and C/T carriers in MCI, and *p* = 0.036 between AD and MCI, Figure 1d, Table 2). However, the low number of individuals in the C/T subgroups (AD, n = 5; MCI, n = 3; controls, n = 4) does not allow for conclusive considerations.

**Figure 3:**
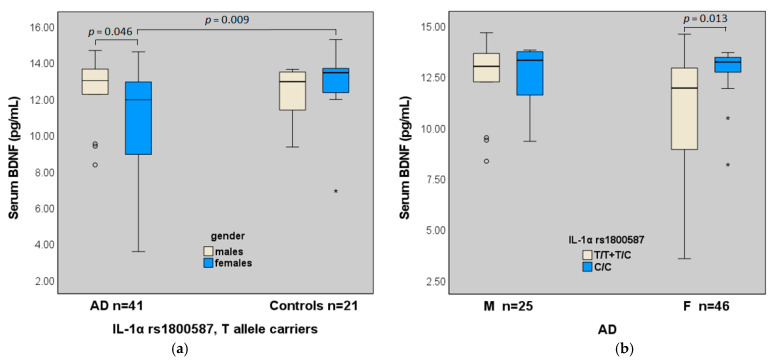
IL-1α rs1800587 genotypes and serum levels of BDNF. (**a**) BDNF levels in carriers of the T allele (T/T, T/C); (**b**) BDNF levels in AD according to gender and IL-1α genotypes.

As for IL-1 family polymorphisms, lower serum BDNF was also associated with the presence of the T allele of IL-1α in AD (T carriers vs. CC homozygous, *p* = 0.040, Table 2), in particular in females, that showed the lowest BDNF levels (n = 26, 10.90 ± 2.84 pg/mL compared with both female controls (n = 14, 12.82 ± 1.91 pg/mL *p* = 0.009), males with AD (n = 15, 12.38 ± 1.86 pg/mL *p* = 0.046) (Figure 3), and female C/C homozygous with AD (n = 20, 12.74 ± 1.32 pg/mL *p* = 0.013).

## 3. Discussion

In this study, gene- and gender-related lower serum levels of BDNF in patients with AD were detected. Serum levels of BDNF were significantly decreased in patients with AD, compared with MCI and the control group. No significant correlations were found between BDNF and positive and negative family history, early vs. late onset disease, risk factors or associated diseases. Data showed a weak correlation between BDNF and age only in controls, confirming previous results of an aging-related reduction in BDNF in serum [26].

Several studies have looked at the serum levels of BDNF in cognitive impairment and AD, with results still controversial. Lower serum levels in AD could be mainly due to both the toxic effects of Aβ accumulation and the reduced physical activity accompanying cognitive decline, as BDNF expression in the hippocampus is increased by exercise [27,28]. In AD, neurotrophins can slow down disease progression and could be the target for novel therapeutic strategies. In physiological conditions, BDNF is mainly synthesized by neurons of the hippocampus and cortex as pro-BDNF and packaged into vesicles for secretion. The main source of circulating BDNF is probably the brain, although other sources should be considered, such as platelets, and endothelial and immune cells [29,30,31]. Decreased serum or plasma levels or reduced BDNF expression in the hippocampus were found in major depression and stress [32,33,34]. Recently, mechanisms that link BDNF regulation, tau phosphorylation, β-amyloid (Aβ) accumulation, and inflammation in AD were discussed [9] as follows: both pro-inflammatory cytokines and the toxic effects of Aβ deposition seem to be associated with alterations of BDNF release. Neurotrophins could be modulated by gene polymorphisms of the same molecule or of their receptors. This is the case for the Val66Met missense variant (G to A at nucleotide 196), the most studied polymorphism of BDNF, often associated with variations in BDNF release. This transition in the pro-domain of the BDNF gene, which produces a Met substitution at codon 66, was involved in AD and developmental cognitive impairment [35], as it affects calcium-dependent BDNF secretion [36].

The BDNF Val66Met variant has a frequency of 20–30% in Caucasians [37]. Results of association studies in AD differ among populations and are still under discussion, as follows: the Val66 allele and Val/Val homozygosity were found more frequently in AD, compared with controls, in Japanese and some Caucasians, while the Met allele was associated with AD severity and progression in other studies [8]. Although most of the studies indicate the Met allele as being associated with cognitive decline in the elderly [38], some studies found the Met variant as protective, while Val/Val homozygous was associated with cognitive decline in the elderly [39,40] and protective in younger individuals [41].

In AD, serum BDNF was significantly lower in females than in males; this gender-related trend of serum BDNF also existed when considering only patients with severe disease, based on the MMSE score. Significantly lower serum levels of BDNF in AD compared to controls were found in carriers of the most frequent BDNF66Val/Val genotype, in particular in the female subset.

In the literature, many observations and associations in AD were gender-specific. Female gender has been recognized as one of the most important risk factors for AD, the second after old age, with about a 2:1 ratio between women and men.

The BDNF expression is differently influenced, in males and females, with many mechanisms, such as stress, gonadal hormones, and epigenetic modulation [42].

A recent review [43] identified the main gender-specific variables associated with AD risk in women, among which the key role is played by the duration of estrogen exposure, including both endogen and exogenous sources (i.e., reproductive lifespan, hormonal therapy, and menopause status). Experimental data demonstrated that mRNA expression of BDNF is regulated by estrogens [44] and decreased levels in the female frontal cortex were observed in aging [45]. Estrogens are also involved in neuroprotection from oxidative stress [46]. The hypothesis is that the drop in estrogens in a postmenopausal period in genetically predisposed women has a greater effect on BDNF transport and reduced expression [47].

These observations raise the hypothesis that estrogen therapy in menopausal women may have protective effects on the onset of AD also by blocking mitochondrial toxicity of Aβ protein and reducing oxidative stress [48]. Although menopausal estrogen supplementation in women currently has no indication in the prevention of AD, recent data have shown positive effects on AD risk and brain health in some conditions [43], and these evidences could allow researchers to identify targets for new personalized treatments based also on gender differences.

Effects of interaction between gender and genotypes on BDNF levels were previously described [49,50,51,52]; in particular, increased BDNF levels were found in male carriers of the Met allele, explained as a hypothetical compensation mechanism for carriers of the less functional and risky BDNF allele [49]. Gender differences in the risk for AD could explain inconsistent or controversial results in the association between AD and BDNF gene polymorphisms. In fact, associations between AD risk and the T allele of the rs56164415 (C270T) [3] or the Met allele of the rs6865 [53] of the BDNF gene were found in females, and female-specific association of BDNFVal66Met and BDNF expression in brain tissues were described [45], indicating BDNF as a female-specific risk gene for AD.

Furthermore, BDNF and neurotrophins can exert opposing region-specific effects and the important role of gene-environment interactions in complex diseases, such as neurodegenerative disorders, is known [33]. Therefore, both gender and genotypes seem to interact to determine serum levels of BDNF, although further factors, such as age or drug therapy, cannot be excluded.

Results of APOE genotyping confirmed the known association of the ε4 allele with AD. In women, APOE-4 was associated with increased Aβ deposition [54] and greater AD risk, although data on APOE–gender interaction are still controversial [55]; as previously described [45], no effects of APOE-4 on BDNF or interactions with gender were detected in the present study.

In addition to APOE and BDNF polymorphisms, we took into examination gene polymorphisms of inflammatory and oxidative stress molecules and their possible effects on serum levels of BDNF and AD risk; however, no significant effects of oxidative stress-related polymorphisms were detected. It has been previously described that increased oxidative stress could induce down-regulation of neurotrophic factors, in a feedback loop that could lead to CNS disorders with cognitive impairment [56]. Oxidative stress and mitochondrial dysfunction are also associated with AD; oxidative stress could be induced by Aβ and iron, [57] and may decrease BDNF expression and promoter activity in vitro [58,59]. Correlations between oxidative damage and Aβ plaques have been detected in both AD and MCI in animal models [14,60]. Gene variants of enzymes, such as SOD (eg the presence of the rs4880 T allele) have been associated with modifications of antioxidant activities, neurodegenerative processes and a greater risk of AD or MCI. As for gender-related findings, it was previously shown that the increased ROS production in mitochondria, induced by Aβ deposition, was lower in females than in males, and this protection from mitochondrial toxicity of Aβ is lost at older ages [48].

Only a few previous studies have examined polymorphisms in oxidative stress genes and AD risk. Associations of a SIRT1 polymorphism with AD were recently validated [25]; however, the SOD2 rs4880 T allele which has been found to increase AD risk and SOD activity was significantly decreased only in combination with the presence of the APOE4 allele [14,61]; other polymorphisms investigated did not show significant results. The present study demonstrated no difference between groups and no association with BDNF levels for oxidative stress-related polymorphisms in AD.

As for IL-1 family genes, the presence of the T allele of the IL-1α rs1800587 was significantly associated with lower serum BDNF in AD, especially in the female subgroup.

The IL-1α rs1800587 is located at position -889 in the promoter region and regulates the IL-1α production [62]. The IL-1α rs1800587 (mutant allele T) was previously indicated as a significant risk factor for AD in Caucasians and different effects of IL-1 polymorphisms were observed according to ethnicity [12]. The presence of the T allele was associated with increased protein levels of IL-1α in other clinical conditions [63]. Therefore, based on these results, an indirect effect of IL-1α polymorphism on BDNF in AD could be assumed. Moreover, the decreased BDNF and increased inflammatory pattern have been already associated with depression [64]. Despite the limitations of a small sample size, this result highlights the opportunity to take into account the potential effects of polymorphisms of inflammatory cytokines when programming novel therapeutic approaches, as some antidepressants may affect inflammatory cytokine activity in the brain [65].

## 4. Materials and Methods

### 4.1. Patients

Patients included in the study were recruited at the Neurology Unit of L’Aquila University and UVA center (Alzheimer Assessment Unit) at San Salvatore Hospital of L’Aquila, and then analyzed in collaboration with the CNR Institute of Translational Pharmacology of L’Aquila (CNR-IFT-L’Aquila Unit). The study was approved by the local ethics committee of Avezzano, Sulmona, L’Aquila ASL (n. 0024209/13). Overall, 110 patients were analyzed, 79 with AD and 31 with MCI (Table 1). The exclusion criteria were represented by the following factors: psychiatric diseases, neurological diseases (including cerebrovascular disease, Huntington’s chorea), neoplasms, uncontrolled diabetes mellitus or arterial hypertension, relevant hepatic/renal/cardiac/pulmonary pathologies, and chronic inflammatory joint disease under NSAIDs. Schooling, family history, and risk factors for AD (head trauma, diabetes, smoking habits, early menopause), and associated diseases were also registered. The MMSE was used to check for cognitive impairment; the score (over or under 20) was used to classify AD severity [5]. A subset of 58 age and gender-matched subjects (age > 55 years), previously analyzed [66] with negative anamnesis for cognitive disorders, were used as controls.

### 4.2. Serum Collection, DNA Extraction and SNP Genotyping

Two milliliters of blood were withdrawn from each subject for the extraction of genomic DNA and the detection of gene polymorphisms, and three milliliters were taken for the separation of the serum for immunoenzymatic assays. Samples were stored at −20 °C until use.

Genomic DNA extraction was performed using commercial kits (QIAamp DNA blood Kit, Qiagen, Germany). Allelic discrimination techniques (TaqMan Single Nucleotide Polymorphisms genotyping assay, Applied Biosystem by Thermo Fisher Scientific Inc., Waltham, MA, USA) were used for the detection of APOE (rs429358 and rs7412), IL-1α (rs1800587), IL-1β (rs1143627), IL-38 (rs6743376), FOXO3A (rs2802292), SIRT3 (rs11555236), GLO1 (rs1049346), and SOD2 (rs4880) in a StepOne real-time PCR equipment (Applied Biosystems, Waltham, MA, USA).

The DNA sequencing of exon 1 and exon 5, which include G196A (Val66Met) and C270T SNPs, respectively, was used for the BDNF gene. The PCR amplifications were carried out using the following primer sets: C270T, 5′-CAGAGGAGCCCGGTGCG-3′ (forward) and 5′-CCTGCACCAAGCCCCATTC-3′ (reverse) [67], G196A, 5′-AAAGAAAGCCCTAACC-3′ (forward), and 5′-TTGTATTCCTCCAGCA-3′ (reverse) [35]. The PCR reactions were performed in a volume of 50 μL containing 250 ng of genomic DNA, 50 nM of each primer, 2.5 mM of each dNTP, 0.3 U of Taq DNA polymerase, and 10× buffer with 2.5 mM MgCl_2_ (Qiagen ).

The PCR conditions were as follows: for Val66Met (G196A), 95 °C for 5min, followed by 32 cycles of 95 °C for 60 s, 55 °C for 30 s, 72 °C for 60 s and final extension at 72 °C for 10 min; for C270T, 95 °C for 5 min, followed by 35 cycles of 95 °C for 60 s, 64 °C for 30 s, 72 °C for 60 s, and final extension at 72 °C for 10 min. After clean up (ExoSAP-IT PCR Product Cleanup kit, Affymetrix, S. Clara, CA, USA), PCR products (Val66Met, 412bp; C270T, 223 bp) were directly sequenced using the Big-Dye Terminator Cycle Sequencing Ready Reaction Kit (Applied Biosystems); capillary electrophoresis was performed on an ABI PRISM 3130 genetic analyzer (Applied Biosystems) and sequences were checked using the Sequencing Analysis and SeqScape software (Thermo Fisher Scientific Inc.).

### 4.3. Enzyme-Linked Immunosorbent Assay for Detection of BDNF

Serum levels of BDNF were detected in 71 patients diagnosed with AD, 31 with MCI, and 32 age-matched controls, using an enzyme-linked immunosorbent assay (ELISA) (ab99978 Abcam, Cambridge, UK). The sensitivity of the test was <80 pg/mL.

### 4.4. Statistical Analysis

Allele and genotype frequencies were calculated for each gene variant. To test the hypothesis of a random union of gametes, the HWE of alleles at individual loci was calculated; haplotypes were estimated using an expectation-maximization (EM) algorithm for multilocus genotypic data when the gametic phase is not known; Arlequin software was used for these analyses [68].

Pearson’s chi-square test was used to evaluate allele and genotype frequencies between groups. To calculate the risk that a particular genotype or allele can represent for the disease, we calculated the odds ratio (OR) and the 95% confidence interval (CI). Statistical significance was assumed with *p* < 0.05. Non-parametric tests (Mann–Whitney U-test, Kruskal–Wallis) were used to evaluate the significant differences in mean values of BDNF concentrations between groups. Statistical analysis was carried out using IBM SPSS Statistics v. 28.

## 5. Conclusions

Differences in immune modulation and inflammatory responses that are found between males and females could affect AD pathogenesis and therapeutic approaches; however, both underlying mechanisms and a possible contribution of autoimmunity in AD are still unknown. A pathogenic link between alterations of circulatory BDNF and autoimmune inflammation was assumed for experimental autoimmune encephalomyelitis, systemic lupus erythematosus, sclerosis, inflammatory bowel disease etc. [69]. Recently, a new model of AD as an autoimmune disease of innate immunity has been theorized, in which Aβ would act as a cytokine-like immunopeptide, the target of an innate autoimmune response that fuels an auto-inflammatory process [70]. In this model, the modulation of endogenous anti-AD molecules would have a central therapeutic role. Since higher BDNF has been correlated with improved cognitive functions, novel promising therapeutic perspectives of direct and indirect targeting of BDNF are being evaluated in order to overcome the reduced BDNF levels in selected AD patients. Neurotrophin-targeted therapies and strategies for BDNF supplementation, such as the use of biodegradable nanocarriers and intranasal delivery, are now undergoing clinical testing [71]. The confirmation of the presence of gender-specific effects on BDNF levels found in this study, the suggestion of no significant effects of oxidative stress-related gene polymorphisms, and the possible contribution of additional genes add further steps in exploring personalized therapeutic strategies.

## Figures and Tables

**Table 1:** Characteristics of patients (AD, MCI) included in the study.

	AD (n = 79)	MCI (n = 31)	*p*
Age at diagnosis, mean ± SD, years	73.79 ± 8.54, 55–92	67.59 ± 9.83, 43–84	0.002
Age at study, mean ± SD, Years ^1^	76.41 ± 8.12, 50–89	68.71 ± 9.68, 43–84	<0.001
Mean period from diagnosis, mean ± SD, years	2.47 ± 2.31, 0–11	1.19 ± 1.55, 0–5	0.009
Gender ^2^ n, %	M = 29, 36.7% F = 50, 63.3%	M = 8, 25.8%; F = 23, 74.2%	ns
Early onset (<65 years), n, %	n = 8, 10.1%	n = 11, 35.5%	0.004
Positive family history, n, %	14, 17.7%	10, 32.3%	ns
Disease duration, years	0–11	0–5	-
MMSE score, (range)	16.68 ± 6.22 (2–27)	24.46 ± 5.27 (6–30)	<0.001
APOE4+ genotypes ^3^ n, %	37, 46.8%	9, 29.0%	ns

^1^ Age at study in controls was 78.22 ± 8.56, 56–100 years, *p* = ns vs. AD, *p* < 0.001 vs. MCI. ^2^ Controls were M = 19, 32.8%; F = 39, 67.2%, *p* = ns vs. AD and MCI. ^3^ APOE4+ genotypes in controls were n = 6, 10.3%, *p* < 0.001 vs. AD, and *p* = 0.025 vs. MCI.

**Table 2:** Serum levels of BDNF in AD and MCI according to gender and genotypes (BDNF Val66Met, C270T; IL-1α rs1800587).

	AD n = 71	MCI n = 31	Controls n = 32	*p* ^1^
Serum BDNF (pg/mL)	11.97 ± 2.24	12.63 ± 2.19	12.88 ± 1.51	0.029 ^2^
*Gender*				
M	12.47 ± 1.68	12.45 ± 2.23	12.56 ± 1.38	ns
F	11.70 ± 2.46	12.70 ± 2.23	13.05 ± 1.58	0.005 ^3^
*BDNF* *Val66Met*				
Val/Val	11.78 ± 2.40	12.63 ± 2.43	13.21 ± 1.19	0.026 ^4^
Met/Met + Val/Met	12.45 ± 1.88	12.53 ± 1.76	12.46 ± 1.80	ns
*BDNF 2* *70C < T*				
C/C	12.10 ± 2.23	12.28 ± 2.26	12.79 ± 1.62	ns
C/T	12.82 ± 1.11	14.74± 0.30	13.31 ± 0.62	0.035 ^5^
*IL-1α rs1800587*				
T/T + T/C	11.44 ± 2.60	12.53 ± 1.93	12.62 ± 1.80	ns ^6^
C/C	12.69 ± 1.35	12.77 ± 2.58	13.38 ± 0.39	ns

Serum BDNF levels were reported as mean ± SD. ^1^ *p* values between AD, MCI, and controls were calculated using the Kruskal–Wallis test. ^2^ *p =* 0.022 between AD and controls; *p* = 0.047 between AD and MCI. ^3^ *p* = 0.002 between AD and controls; *p* = 0.031 between AD and MCI. ^4^ *p* = 0.010 between AD and controls. ^5^ *p* = 0.021 between C/C and C/T genotypes in MCI; C/T carriers: *p* = 0.036 between AD and MCI. ^6^ *p* = 0.040 between C/C and T/T + T/C genotypes in AD.

## Data Availability

The data that support the findings of this study are available within the article and its supplementary information or from the corresponding author on reasonable request.

## Supplementary Materials

The following supporting information can be downloaded at: https://www.mdpi.com/article/10.3390/ijms232314599/s1.
