# Supplementary material for: Gene- and Gender-Related Decrease in Serum BDNF Levels in Alzheimer’s Disease
Source: Int J Mol Sci. 2022 Nov 23;23(23):14599. doi: 10.3390/ijms232314599 (PMC9740390; doi:10.3390/ijms232314599)
Supplement: Supplementary file 1 [file ijms-23-14599-s001.zip › ijms-2029746-supplementary.pdf]

Table S1. APOE allele and genotype frequencies in AD, MCI and controls.

	AD		MCI		Controls	
**genotypes**	**n**	**%**	**n**	**%**	**n**	**%**
ε3/ε3	39	49.4	17	54.8	44	75.9
ε3/ε4	32	40.5	7	22.6	5	8.6
ε2/ε3	2	2.5	5	16.1	7	12.1
ε2/ε4	2	2.5	2	6.5	1	1.7
ε2/ε2	1	1.3	0	0	1	1.7
ε4/ε4	3	3.8	0	0	0	0
**alleles**						
ε2	6	3.8	7	11.3	10	8.6
ε3	112	70.9	46	74.2	100	86.2
ε4	40	25.3	9	14.5	6	5.2

APOE genotype distribution between groups: p=0.001; ε4+ between groups: p<0.001.

Table S2. IL-1α-IL-1β-IL-38 estimated haplotypes (f>0.05) in AD, MCI and controls.

**IL-1A-IL-1B-IL-38 haplotypes**	**AD**		**MCI**		**Controls**	
	n	f	n	f	n	f
CCA	21	0.137	12	0.190	19	0.167
CCC	15	0.096	0	0.000	5	0.040
CTA	42	0.271	20	0.324	41	0.354
CTC	22	0.145	8	0.130	18	0.155
TCA	12	0.078	2	0.035	5	0.043
TTA	30	0.196	11	0.175	14	0.117
TTC	12	0.076	9	0.143	14	0.124

f=haplotype frequency; p=ns.

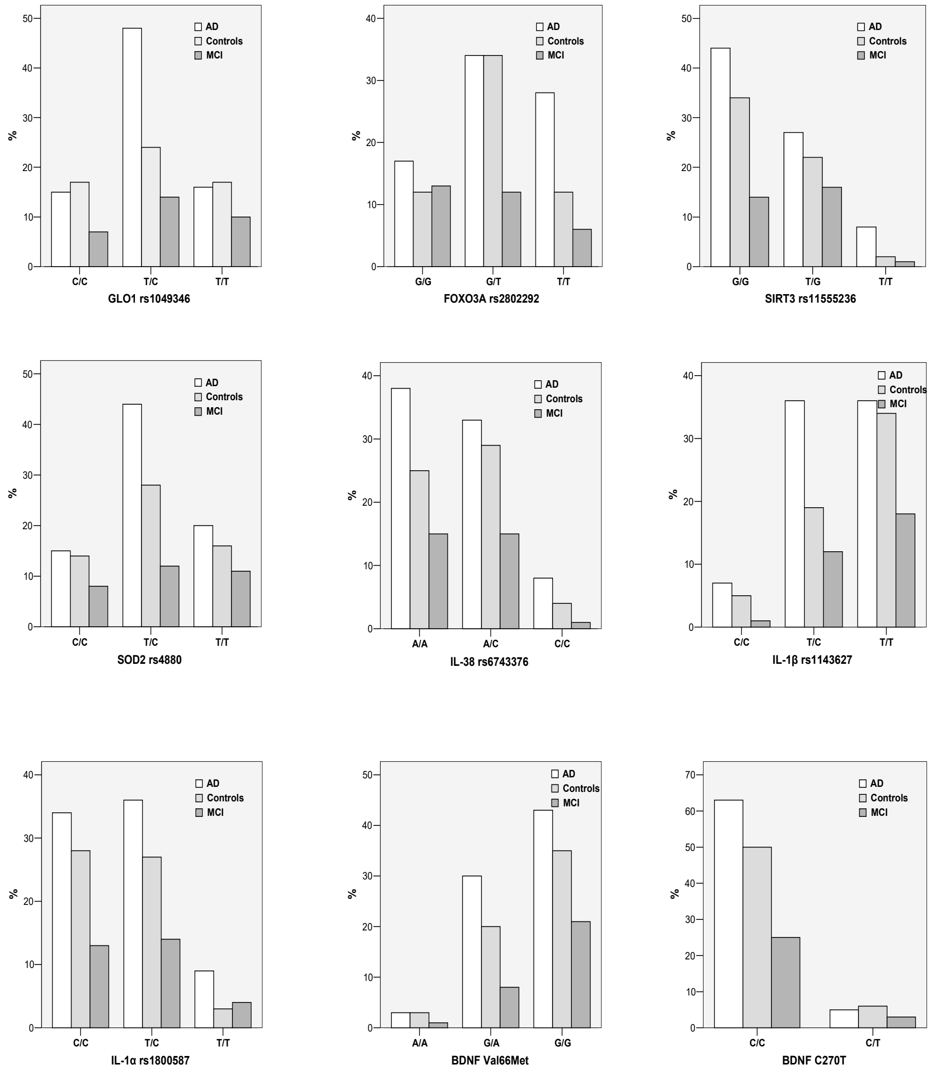

Figure S1. Genotype frequencies of the assayed polymorphisms in AD, MCI and controls (p=ns, Kruskal-Wallis test).
